# A Technique to Improve the Success of Stent Placement for High‐Level Malignant Biliary Obstruction Through PTCD Tract: A Case Report

**DOI:** 10.1155/carm/8550138

**Published:** 2026-04-04

**Authors:** Xu Lan, Yue-Xiao Lin, Yun-Xiao Wang, Yi-Fei Dong, Jia-Jia Duan

**Affiliations:** ^1^ Department of General Surgery, Beijing University of Chinese Medicine Third Affiliated Hospital, Beijing, China, bucm.edu.cn; ^2^ Department of Cardiology, Beijing University of Chinese Medicine Third Affiliated Hospital, Beijing, China, bucm.edu.cn

**Keywords:** case report, high-level malignant biliary obstruction, stent placement, vascular sheath

## Abstract

For patients with high‐level malignant biliary obstruction—confirmed following initial percutaneous transhepatic cholangiography drainage (PTCD) performed for preoperative bilirubin reduction and subsequently determined to be unresectable—biliary stent placement via the existing PTCD tract represents a straightforward, minimally invasive, and clinically feasible therapeutic strategy. However, the procedure may be technically challenging if the catheter tract is tortuous. This case report describes a refined interventional technique to overcome such limitations. A 56‐year‐old man was admitted due to right upper quadrant abdominal pain and progressive jaundice involving both the skin and sclera. Initial PTCD was performed to achieve rapid biliary decompression and reduce serum bilirubin levels. Subsequently, comprehensive imaging and histopathological evaluation established the diagnosis of unresectable hilar cholangiocarcinoma with intrahepatic and multiple osseous metastases. Given the patient’s unresectability and need for durable biliary drainage, stent placement through the preexisting PTCD tract was selected as the optimal palliative intervention. During the procedure, a hydrophilic guidewire was advanced through the indwelling PTCD catheter into the duodenum through the obstructed hepatic portal bile duct and common bile duct; the catheter was then withdrawn over the wire, and a vascular sheath (size similar to the original PTCD tract) was inserted coaxially over the guidewire. This maneuver effectively straightened the access pathway and provided mechanical support for subsequent device delivery. A self‐expanding metallic biliary stent was then successfully deployed along the PTCD tract. Postprocedural cholangiography confirmed complete stent apposition, unobstructed bile flow into the duodenum, and absence of procedural complications. This case demonstrates that the judicious use of a vascular sheath during PTCD tract–based stent placement enhances procedural safety, efficiency, and technical success—while minimizing procedural complexity and patient discomfort—and represents a practical, reproducible refinement for biliary stent deployment in anatomically challenging high‐grade malignant biliary obstruction.

## 1. Background

High‐level biliary obstruction refers to an obstruction occurring in the hepatic hilar bile duct region above the common hepatic duct, often caused by factors such as choledocholithiasis, inflammatory strictures, and neoplastic lesions. This obstruction disrupts the physiological passage of bile into the duodenum, thereby precipitating a constellation of severe gastrointestinal manifestations, including right upper quadrant abdominal pain, obstructive jaundice, fever, nausea, and vomiting. In the absence of prompt biliary decompression, life‐threatening complications may ensue, notably ascending cholangitis and progressive hepatic failure.

For patients with high‐level biliary obstruction, particularly when a definitive diagnosis and corresponding treatment plan remain undetermined, prompt and effective biliary decompression is crucially important [1]. Among the available treatment options, percutaneous transhepatic cholangiography drainage (PTCD) has emerged as a well‐established, minimally invasive intervention, playing a pivotal role in reducing jaundice and alleviating associated symptoms [2].

PTCD guided by digital subtraction angiography (DSA) is commonly employed in clinical practice. However, this procedure requires the placement of an external biliary drainage catheter, resulting in substantial external bile loss, which significantly impairs gastrointestinal digestion and nutrient absorption. Moreover, potential complications include catheter dislodgement, infection, and hemorrhage [2, 3].

Therefore, when a malignant tumor has been definitively diagnosed and surgical resection is not feasible, placement of a biliary stent can maintain biliary patency, ensuring adequate bile drainage into the duodenum, and thereby better preserving the body’s native physiological function [4]. Nevertheless, placing a biliary stent through a PTCD tract in cases of high‐level malignant biliary obstruction is not straightforward.

Thus, the purpose of this case report is to introduce a straightforward and effective technique for a successful biliary stent placement through an existing PTCD tract in patients with high‐level malignant biliary obstruction.

## 2. Case Report

A 56‐year‐old man was admitted due to right upper quadrant abdominal pain and progressive jaundice involving both the skin and sclera lasting for half a month, without fever, nausea, or vomiting. He had a past history of hypertension, hyperlipidemia, coronary heart disease with coronary stent implantation, and Type 2 diabetes. The remainder of the review of systems revealed no obvious abnormalities. The patient had a personal history of long‐term smoking. There was no family history of tumor. He had not taken any medications recently. He denied alcohol use or use of other substances.

Physical examination on admission showed the following: body temperature, 36.3°C; heart rate, 96 beats per minute; blood pressure, 116/82 mmHg; respiratory rate, 20 breaths per minute; and pulse oximetry, 99%. Cardiac and pulmonary examinations revealed no abnormalities. Specialized examination showed jaundice of the skin and sclera, deep tenderness in the right upper abdomen, and no rebound tenderness or muscle rigidity. The remainder of the abdominal examination was normal. Laboratory examination showed the following: white blood cell count, 6.86 × 10^9^/L; neutrophils, 77.5%; hemoglobin, 141 g/L; platelets, 279 × 10^9^/L; C‐reactive protein, 21.3 mg/L; alanine aminotransferase, 78 U/L; aspartate aminotransferase, 43 U/L; alkaline phosphatase, 311 U/L; γ‐glutamyltransferase, 231.4 U/L; total bilirubin, 830.3 μmol/L; direct bilirubin, 415.7 μmol/L; albumin, 33.5 g/L; carcinoembryonic antigen, 37.24 ng/mL; and carbohydrate antigen 199 > 10,000 U/mL. Electrocardiography and echocardiography were normal. Abdominal contrast‐enhanced CT scan revealed biliary obstruction caused by hilar bile duct occupation and a mass in hepatic segment VI.

**FIGURE 1:**
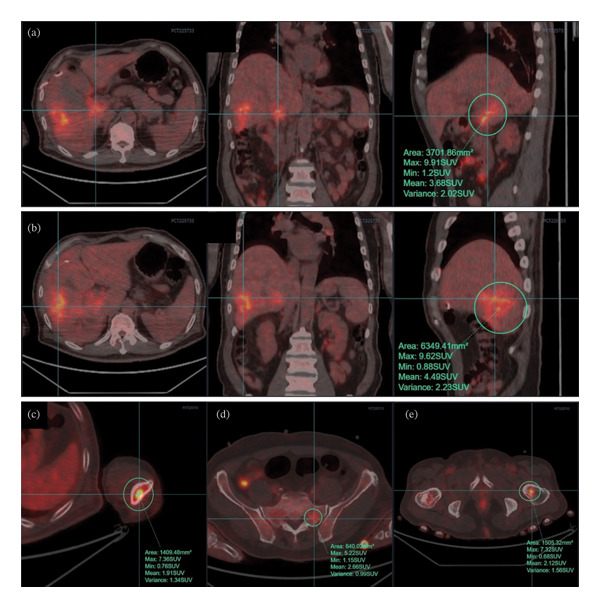
^18^F‐FDG PET/CT revealed hilar cholangiocarcinoma with intrahepatic and multiple osseous metastases. (a) The hilar cholangiocarcinoma demonstrated a standard uptake value (SUV) of 9.91. (b) The intrahepatic metastasis demonstrated an SUV of 9.62. (c–e) The multiple osseous metastases demonstrated SUVs ranging from 5.22 to 7.36.

**FIGURE 2:**
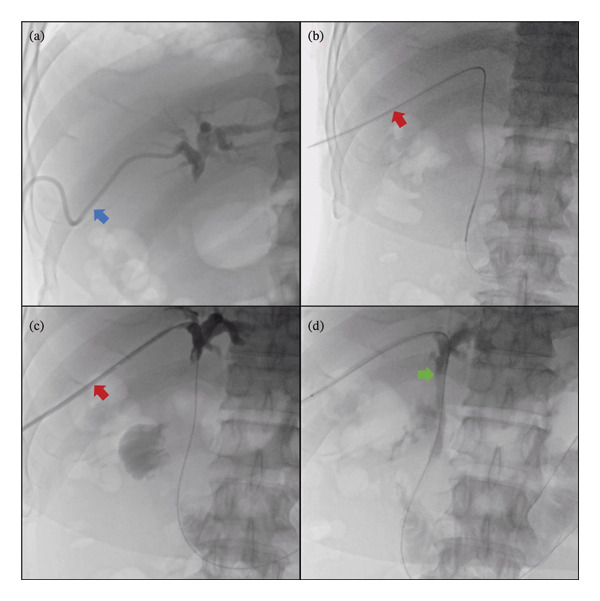
Successful stent placement into the high‐level common hepatic duct was achieved with vascular sheath assistance. (a) Cholangiography through the original external drainage PTCD catheter revealed a tortuous original tract and a slightly higher intrahepatic duct position, with occlusion of the hilar bile duct (blue arrow). (b) After vascular sheath insertion, the tract became straighter (red arrow), providing effective support and facilitating catheter and guidewire manipulation. (c) Cholangiography through the vascular sheath confirmed high‐level common hepatic duct obstruction. (d) A biliary stent (green arrow) was placed through the vascular sheath; poststent cholangiography demonstrated unobstructed common bile duct flow, with contrast medium smoothly entering the duodenum.

In order to prevent severe complications such as hepatic failure and hemorrhage, DSA‐guided PTCD catheter placement was initially performed at a higher puncture site to avoid traversing the tumor. Subsequent β‐2‐[18F]‐fluoro‐2‐deoxy‐D‐glucose (^18^F‐FDG) positron emission tomography/computed tomography (PET/CT) (Figure 1) and pathological examination of hepatic mass biopsy confirmed the diagnosis of hilar cholangiocarcinoma with intrahepatic and multiple osseous metastases. As surgical treatment was not feasible, biliary stent placement via the PTCD tract was performed to ensure biliary patency and preserve the normal anatomical and physiological integrity of the digestive tract. The procedure was as follows: First, cholangiography through the original external drainage PTCD catheter revealed a tortuous original tract and a slightly higher intrahepatic duct position, with occlusion of the hilar bile duct (Figure 2(a)). Second, a guidewire was inserted along the PTCD catheter; the catheter was then withdrawn, and a vascular sheath was advanced over the guidewire. The original tract straightened after the insertion of the vascular sheath (Figure 2(b)). Third, under support from the vascular sheath, a contrast catheter and guidewire successfully traversed the occluded hilar bile duct segment and entered the duodenum (Figure 2(c)). Fourth, a biliary stent was successfully placed across the occluded hilar bile duct segment through the vascular sheath; cholangiography confirmed patency of the common bile duct and smooth flow of contrast medium into the duodenum (Figure 2(d)). No complications or adverse events occurred.

Subsequently, after serum bilirubin levels normalized, the patient began scheduled chemotherapy.

## 3. Discussion

In this case report, we successfully achieved stent placement for high‐level malignant biliary obstruction by inserting a vascular sheath through the PTCD tract.

The key technical consideration of this method is the selection of a vascular sheath whose diameter is similar to that of the indwelling PTCD catheter. A sheath with a smaller diameter may compromise tract stability and intraluminal support, whereas one with a larger diameter increases the risk of hepatic hemorrhage. Moreover, a standard nonpeel‐away vascular sheath is sufficient to meet the procedural requirements, and tract dilation is generally unnecessary prior to sheath placement.

The advantages of this technique are primarily as follows: First, operational simplicity: the procedure is technically straightforward and does not require complex instrumentation [5]. Second, enhanced guidewire support: due to the stiffness of malignant tumor tissue, high‐level biliary obstruction is challenging to negotiate. Therefore, the guidewire requires sufficient support to traverse the stricture; this is also the primary cause of stent placement failure via the PTCD tract. Placing a vascular sheath provides adequate guidewire support and effectively shortens the working distance, thereby facilitating subsequent steps. Third, minimal patient discomfort: the procedure can be performed under local anesthesia alone, avoiding additional pain for the patient [6]. Fourth, for high‐level malignant biliary obstruction, compared with alternative stent placement methods, such as endoscopic retrograde cholangiopancreatography (ERCP), this approach avoids duodenal papillotomy and its associated complications, including bleeding, perforation, and recurrent reflux cholangitis [7, 8].

The potential complications associated with this technique include hemorrhage and hepatic rupture [9]. However, given that the selected vascular sheath has a diameter similar to that of the original PTCD catheter, and the procedure is performed under DSA guidance with the sheath advanced over the guidewire, the risk of these complications is minimal.

In conclusion, although several techniques are available for successful biliary stent placement in cases of malignant biliary obstruction, utilizing the pre‐existing PTCD tract is the simplest and most efficient approach. As a practical tip, the appropriate use of a vascular sheath can facilitate subsequent procedures and represents a technical refinement for biliary stent placement.

## Funding

No funding was received for this research.

## Disclosure

This study has not been previously presented at any conferences or in abstract form.

## Consent

Written informed consent was obtained from the family members of the patient.

## Conflicts of Interest

The authors declare no conflicts of interest.

## Data Availability

The data that support the findings of this study are available on request from the corresponding author. The data are not publicly available due to privacy or ethical restrictions.
